# Seeing through cells: Rapid measurement of intracellular target proteins

**DOI:** 10.3389/fbioe.2022.996224

**Published:** 2022-10-03

**Authors:** Per-Olof Larsson, Nelida Leiva Eriksson

**Affiliations:** ^1^ Division of Pure and Applied Biochemistry, Lund University, Lund, Sweden; ^2^ Division of Biotechnology, Lund University, Lund, Sweden

**Keywords:** transparent cell suspensions, refractive index, hemoglobin, intracellular proteins, BSA, iodixanol, FicollTM

## Abstract

We have studied a method for making microbial cells transparent by immersing them in a solution with a high refractive index (RI). When the RI of the solution was matching that of the cells, light scattering was greatly diminished (by a factor of up to about 100) and the cell suspension became transparent, facilitating the spectrophotometric determination of intracellular compounds such as hemoglobin. We investigated the properties of several compounds such as sucrose, glycerol, bovine serum albumin, Ficoll^TM,^ and iodixanol (Optiprep^TM^), each with advantages and disadvantages. Particularly good overall properties were found for iodixanol at a concentration of around 36% (w/v) and bovine serum albumin at a concentration of about 30% (w/v). By using this RI-matching principle the production of intracellular compounds can easily be followed in near real-time during fermentation processes. For example, some conditions for producing plant hemoglobin in *Escherichia coli* were conveniently determined without the need of any cell disintegration or product purification.

## Introduction

When developing and optimizing a bioprocess method, it is of great importance to determine the concentration of the target product within the cell in the most direct way. Currently used methods rely mainly on cell lysis to release the product, centrifugation steps, photometric quantification or labelling of target products which requires additional design. If many parameters need to be checked, such an optimization process may become quite taxing. Furthermore, the necessity of lysing and subsequent centrifugation steps may lead to losses of the target protein, resulting in less accurate results.

The most direct way to follow the progress during protein production optimization would be to determine the concentration of the target product within the intact cells without having to damage the cell membrane. However, suspensions with intact cells cannot be analysed with standard spectrophotometers since they scatter light very strongly, making, for example, a suspension containing 10 mg/ml of cells totally opaque. There are specialized spectrophotometers available, which use an integrating sphere together with dedicated software. However, such equipment is not usually available in a standard laboratory, so an alternative approach for spectrophotometric analysis is desirable.

Here we have studied a procedure for a fast, direct measurement of intracellular compounds that should be valuable when optimizing the production of proteins. The procedure is based on a long well known, but in this context, overlooked principle, namely refractive index (RI) matching (Barer et al. 1953; Barer 1955; Liu et al., 2016; Gul et al. 2021). The cells to be analyzed with respect to their content of target protein are suspended in a medium with a RI that matches their average RI. In that way, light scattering is largely removed, the cells become transparent, and the concentration of intracellular compounds can be determined spectrophotometrically in a direct way. This RI matching principle is fundamentally simple and has been described many times, e.g., for the determination of RI of minerals (Larsen and Berman, 1934) and of microbial cells (Bateman et al. 1965), and for making chromatographic support materials (Larsson, 1983) and microbial cells (Barer and Joseph 1954; Barer and Joseph, 1955a; Barer 1955; Barer and Joseph 1955b) as well as living animal cells and tissues transparent (Boothe et al. 2017).

With this background, we have undertaken to look at several media with a suitably high RI and see how they will practically perform as matching agents for e.g., *E. coli* cells. Factors we have considered are the molecular weight/size, viscosity, absorbance, ease of handling and cost of the RI enhancer. We have also briefly considered the initially somewhat unexpected change of cellular RI with fermentation time.

## Materials and methods

### Special chemicals and cultivation of cells

Sources for special chemicals and cultivation protocols are given in Supplementary Material: 1. Special chemicals and 2. Cell cultivation. The *Escherichia coli* BL21-DE3 cells expressing hemoglobins were cultivated as described by Leiva Eriksson et al., 2019. In short, cells containing the gene of a phytoglobin in an expression vector were grown in sterile TB medium containing 100 mg/ml carbenicillin. The gene expression was induced after 3 h of growth by adding 0.5 mM IPTG (isopropyl β-D-thiogalactoside) and 0.4 mM 5-ALA (5-aminolevulinic acid). Harvesting of the cells was done around 16 h after induction.

### Preparation and handling of viscous stock solutions

Some of the solutions used for RI matching were very viscous (e.g., bovine serum albumin, BSA) and Ficoll^TM^ 400) and were more easily prepared on a weight basis. For instance, a 35% w/w BSA stock solution was prepared by mixing 17,5 g BSA with 32.5 g of water in a 100 ml stoppered flask which was placed on a rocking table for a couple of hours until a homogeneous solution was obtained. This solution had a density of 1.101 g/ml at room temperature (measured by pycnometer or calculated from the apparent specific volume for BSA (0.739 ml/g) (Chalikian et al. 1996). By knowing the BSA content of the stock solution and its density, it was straightforward to obtain working solutions with any lower BSA content expressed as weight/weight percentage or weight/volume percentage (Supplementary Material: 4. Preparation and use of RI enhancer solutions).

When carrying out experiments with viscous solutions ordinary pipettes could not be used given that the solutions would only be partially expelled when emptying the pipette. Instead, positive displacement pipettes were used. Weighing the viscous solutions is an alternative procedure but slow and inconvenient.

### Spectrophotometric measurements of cell suspensions

The spectrophotometric measurements were carried out according to a Standard procedure, where the cells were mixed with a RI enhancer solution having suitable concentration followed by spectrophotometric measurements. An Alternative procedure (sequential addition) was convenient to use when not knowing the optimal concentration of the RI enhancer. Here the cells were mixed with a strong RI enhancer solution and OD600 measured. Subsequently, a small, pre-calculated amount of water/buffer was added to give a new, known concentration of the RI enhancer. The cuvette content was mixed and OD600 again recorded and so on.

#### Standard procedure

The bacterial cell suspension (0.50–2.00 ml) was centrifuged (2.5 min; 5,000 x g). The supernatant was carefully removed, and the pellet suspended in 1.5 ml wash buffer (0.05 M Tris-HCl, pH 8.0). The cells were again spun down and the wash solution carefully removed. The pellet was subsequently suspended in 0.50 ml RI enhancer, e.g., 30% (w/v) BSA or 36% (w/v) iodixanol using a vibromixer (Lab-Line Instruments, IL, United States). It was important to control that the pellet had been completely resuspended. The wash step in this procedure can be omitted if desired.

The cell suspension was then immediately transferred to a cuvette (1.5 ml PMMA plastic cuvettes) for spectrophotometric measurements. To allow any gas bubbles to disappear from viscous solutions a few minutes waiting time was observed. Mixing protocols and a variant of this procedure is given in Supplementary Material: 4. Preparation and use of RI enhancer solutions.

The spectrophotometer used was a standard diode array instrument (Lightwave II from Biochrom Ltd, UK). As a reference/blank, the corresponding RI enhancer solution was used. By having the cuvette properly height adjusted a sample volume of 0.4 ml was sufficient. With proper cuvettes even smaller volumes could be managed.

#### Alternative procedure (sequential addition)

To determine the optimum concentration of a RI enhancer, it was convenient to carry out all mixing operations in the measuring cuvette, using a sequential dilution approach. A cell suspension, e.g. 100 uL, was carefully mixed in the cuvette with a concentrated RI enhancer solution, e.g. 400 uL 60% (w/v) iodixanol solution. The resulting 48% solution was measured to give OD600. Subsequently, a small, pre-calculated amount of water/buffer was added to give a new, known concentration. The cuvette content was mixed and OD600 again recorded and so on. This sequential dilution initially diminished the OD600, the wanted minimum was reached, and then the OD600 again started to increase. Mixing protocols are given in Supplementary Material: 4. Preparation and use of RI enhancer solutions.

The mixing in the cuvette is not a trivial operation since it involves the difficult mixing of a viscous component (BSA) or a high-density component (iodixanol) with low density/low viscosity water. A preferred way was to use a flat spatula and also to use a bright light source in order to observe any remaining Schlieren patterns indicating insufficient mixing which would lead to misleading results.

## Results and discussion

**FIGURE 1:**
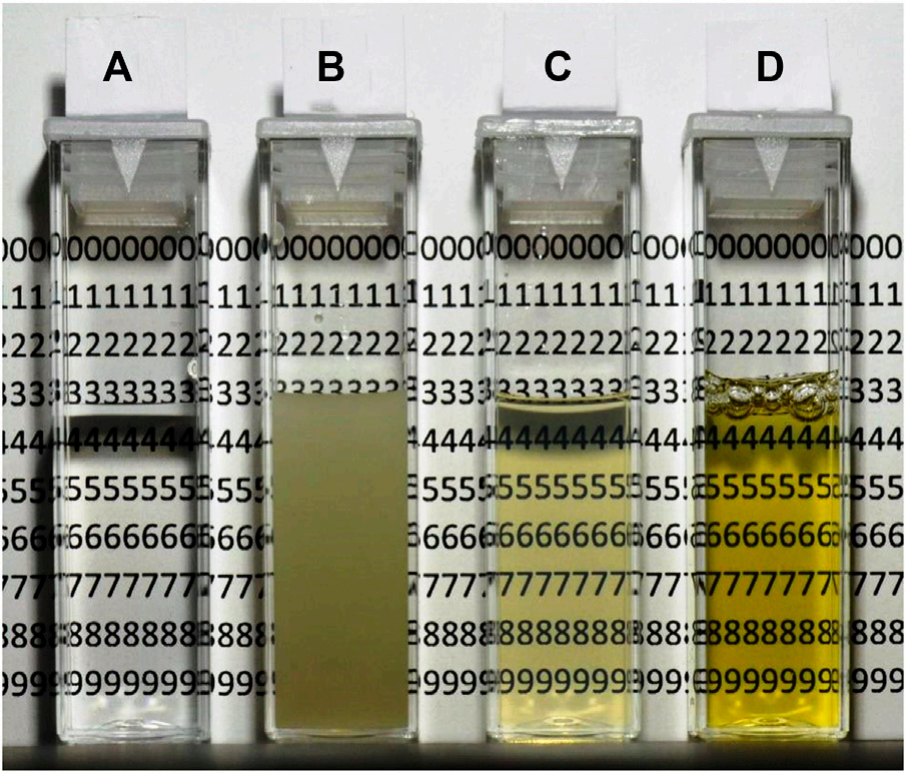
Transparency of *E. coli* suspensions in solutions with high refractive index (RI). **(A)** Reference cuvette with water, no cells. **(B)** Cells in water. **(C)** Cells in 36% w/v iodixanol. **(D)** Cells in 30% w/v bovine serum albumin (BSA). The cell concentration is the same in cuvettes **(B–D)**, about 11 mg (wet weight)/ml.

The possibility of rapidly measuring intracellular compounds without prior cell disintegration, centrifugation and pre-purification is very attractive. This is particularly so if a fermentation protocol is developed where many samples are necessary to process and where the time aspect is important. The method suggested here, namely, to immerse the cells in a medium with the same RI as the average RI of the cells is based on a well-established but underused knowledge, as pointed out above. An immediate appreciation of the potential of the method can be gathered from Figure 1 that shows the light scattering effects of *E. coli* cells in solutions with two very effective RI enhancers, namely iodixanol (C) and BSA (D). As is immediately obvious the cell suspension without enhancers (B) is very opaque and impossible to use to obtain meaningful spectra (OD_600_ = 11). Iodixanol and BSA on the other hand gave transparent solutions, almost in parity with the reference (A) containing water and no cells.

Below we compare the RI enhancers with respect to their molecular size, absorbance properties, and their ability to make cells transparent as well as discuss parameters such as viscosity and ease of handling. The fact that cells change their average RI with fermentation time is also considered. A comparison between the standard way of assaying Hb and the RI enhancer method is carried out and, finally, the practical use of the procedure is illustrated by the monitoring of a fermentation producing plant Hb in *E. coli* cells.

### Refractive index (RI) enhancers make cells transparent

A series of experiments were carried out to find the optimum concentration for several RI enhancers, i.e., when the light scattering of suspended cells becomes minimal. The substances chosen were known to be compatible with various types of cells and known to be able to form concentrated aqueous solutions, which is necessary in order to obtain a RI high enough to match that of bacterial and other cells. Based on very early reports in this area, BSA was a natural first choice (Barer et al. 1953). Also, Ficoll^TM^ and sucrose, which are often being used in high concentration to handle cells and proteins in density centrifugation qualified. Finally, iodixanol (OptiPrep^TM^, Visipaque^TM^) was selected, known to be compatible with bacterial and animal cells (Boothe et al. 2017) and used for density gradients as well as an X-ray contrast medium.

**FIGURE 2:**
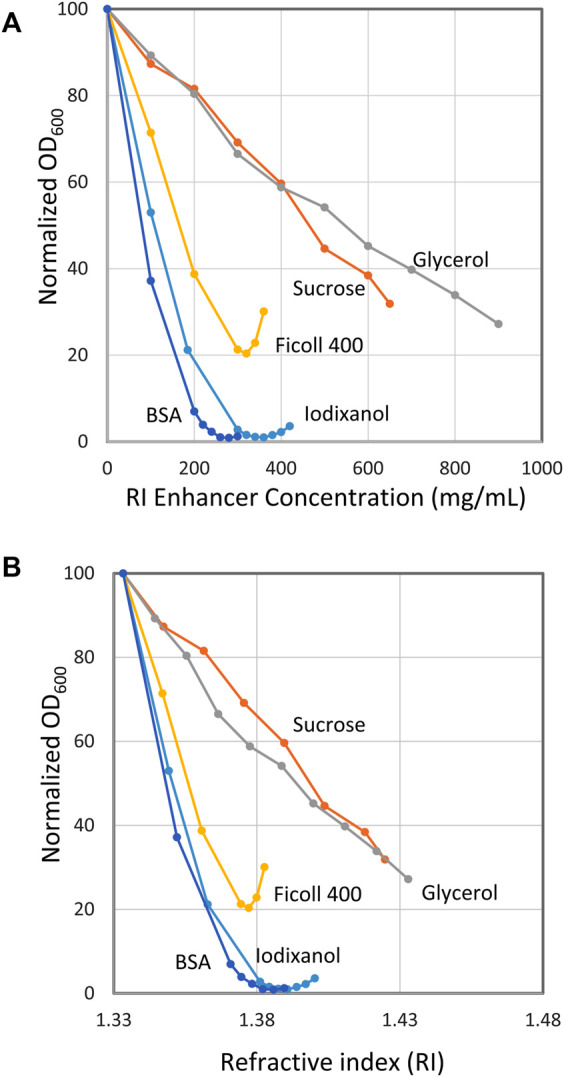
Effect of refractive index (RI) enhancers on the optical density of suspensions of *E. coli*. **(A)** Optical density as a function of enhancer concentration. **(B)** Optical density as a function of RI. The x axis gives the normalized optical density (OD_600_ = 100 at 0% RI enhancer concentration). Bacteria stock suspensions were mixed with RI enhancer and buffer to give a final volume of 500 µL with the indicated RI enhancer concentration. The stock bacteria suspensions used for the different curves in the diagram had an OD_600_ of 20–42 (extrapolated). The stock suspension volumes (5–100 µL) were chosen to give readings below 1.5 to secure optical linearity.

Figure 2A shows the results with *E. coli* suspended in BSA, iodixanol, Ficoll^TM^400, sucrose and glycerol. The graph gives the normalized light scattering (OD_600_) as a function of RI enhancer concentration (mg/ml). As could be expected from the qualitative results in Figure 1, excellent quantitative data were obtained for both BSA and iodixanol. In fact, the minimum light scattering was in both cases below 1% of the maximum scattering obtained in the absence of RI enhancer. The curve around the minimum is rather flat for both BSA and iodixanol, making it not too important to precisely hit the minimum unless the very best transparency is desired. Thus, 30% (w/v) for BSA and 36% (w/v) for iodixanol were chosen as suitable standard concentrations for further experiments. Ficoll^TM^400 diminished the light scattering considerably but not as impressively as BSA and iodixanol and gave a minimum at approximately 20% of maximum scattering. Sucrose and glycerol also diminished scattering but never reached a satisfactory minimum despite high concentrations of the respective enhancer.

It is rewarding to also look at Figure 2B, which is the same as Figure 2A with the exception that here the light scattering is plotted against the RI. The minimum for BSA, iodixanol and Ficoll^TM^ 400 is reached at a RI value approximately the same as is ascribed to *E. coli* cells, 1,388 (Liu et al. 2014). This implies that these compounds neither interfere with nor change the intrinsic RI of the bacterium. For BSA and Ficoll^TM^400, this is a very reasonable assumption considering that they are large molecules (66 and 400 kDa) and therefore will not have access to the inner parts of the bacterium through its encapsulating membranes, provided the membranes are intact. Also, the protein nature of BSA is principally quite similar to that of the cell interior with respect to pH, charged molecules and osmotic properties, a situation that further diminishes the influence of the medium on the cells (Barer and Joseph 1955a). The situation might be expected to be different for iodixanol, which is a smaller molecule (1,550 Da), and therefore will exhibit a higher osmotic pressure than BSA. Apparently, this effect is not strong enough to cause any appreciable increase of the *E. coli* RI. The fact that iodixanol is used for handling animal cells without any adverse effect is in line with this view (Boothe et al. 2017). As can be seen, Ficoll^TM^400 gave a less pronounced minimum at a slightly lower RI than, for example, BSA. It was also noted that small, non-intended variations in the procedures used could shift the minimum, an observation that together with its high viscosity made Ficoll^TM^400 a less attractive RI enhancer.

For sucrose and glycerol, the situation is quite different. Both being low molecular weight compounds in high concentration, they will exhibit a very high osmotic pressure leading to osmotic shock and immediate cell shrinkage with consequential increase of the internal RI (Koch, 1984; Pilizota and Shaevitz 2012). Furthermore, such low molecular weight substances may also pass the cell membranes, additionally increasing the RI of the cell interior (replacing water). Increasing the medium concentration further just leads to a higher internal RI and there will never be an equilibrium between the cell and its surroundings (Barer and Joseph 1955a). Consequently, no minimum is seen in the curve. Still, at the highest glycerol concentration tested, 900 mg/ml, the light scattering has diminished to 25% of the original.

30% (w/v) BSA and 36% (w/v) iodixanol were selected as standard concentrations for making cells translucent. Centrifuged cell pellets were suspended directly in these media or, alternatively, suspensions of cells were mixed with more concentrated media.

It was noted that these standard concentrations were not perfect for all situations. For example, “older cells” (several hours of cultivation) showed minimum scattering at a higher RI enhancer concentration than “younger ones” (few hours of cultivation). Illustrative data are presented in Supplementary Material: 5. Cultivation time–shift of minimum). The data refers to a 4–19 h cultivation time and the concentration needed for minimum scattering increased from about 28% to about 31% for BSA and from about 33% to about 40% for iodixanol. The increase probably reflects inherently denser cells and perhaps also a higher permeability, suggested by the more pronounced increase with the low molecular weight iodixanol.

Other factors might conceivably also influence the percentage of enhancer needed to obtain the minimum, e.g., strain, composition of the fermentation broth, temperature, osmotic pressure, etc. However, unless maximum translucency is necessary, the same standard concentration is convenient to use.

### Absorbance and viscosity properties of refractive index (RI) enhancers

Ideally, RI enhancers should have a very low absorbance in the part of the spectrum where the target molecules absorb. This is accentuated by the fact that the enhancers must be used in very high concentration such as 300 mg/ml to prevent cells from scattering light as was shown above (Figure 2A). Thus, even a low extinction coefficient may result in a prohibitively high background absorbance.

**FIGURE 3:**
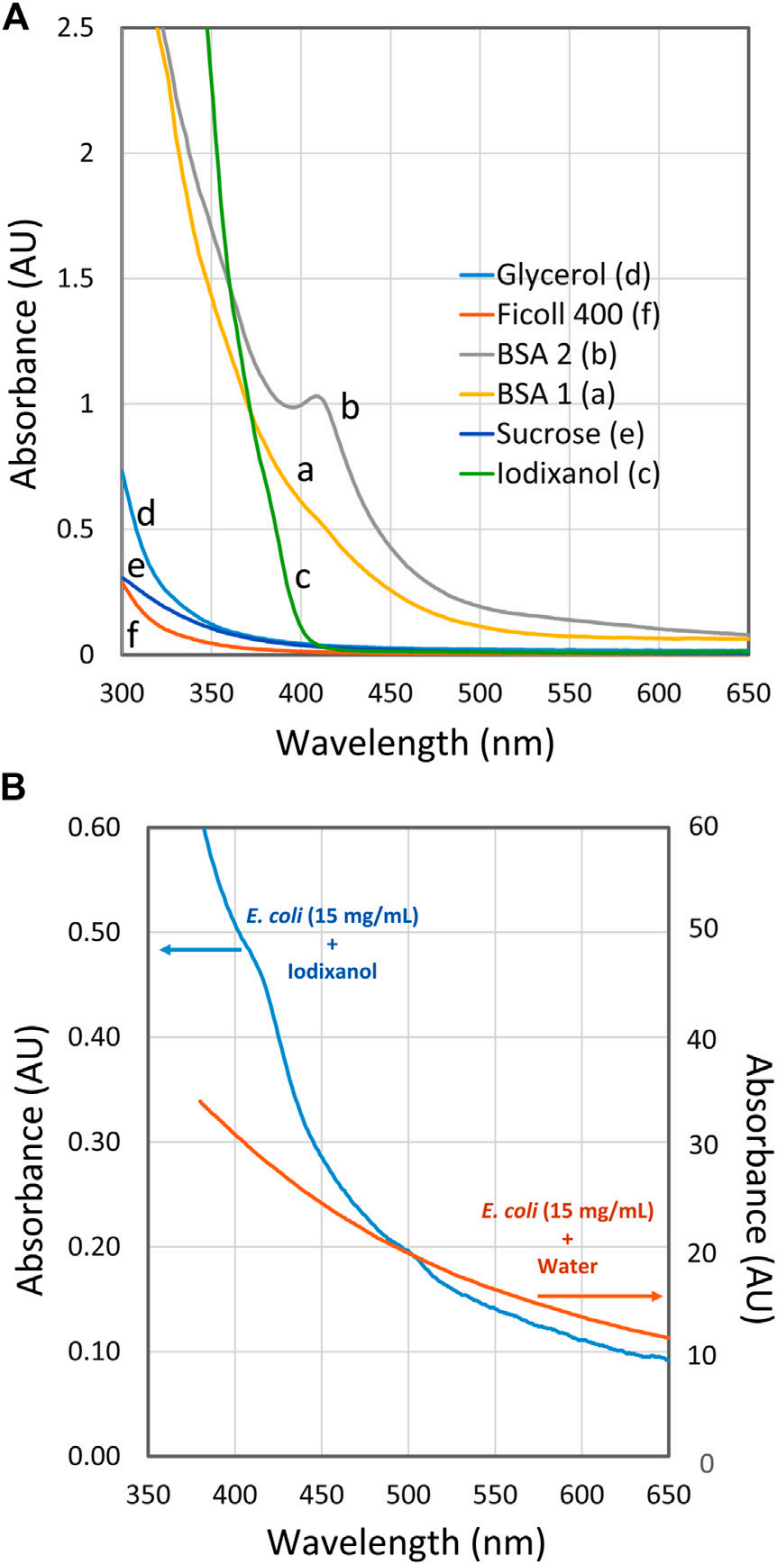
**S**pectra of RI enhancers at high concentration. **(A)** Spectra of refractive index (RI) enhancers: BSA 1 **(a)**, BSA 2 **(b)**, iodixanol **(c)**, glycerol **(d)**, sucrose **(e)** and Ficoll^TM^400 **(f)** with water as reference. The concentration of the respective enhancers was close to the concentrations giving minimum scattering as shown in Figure 2, i.e., 300 mg/ml for BSA and Ficoll^TM^, 360 mg/ml for iodixanol, 550 mg/ml for sucrose and 900 mg/ml for glycerol. **(B)** Spectra of *E. coli* suspension (OD_600_ = 13.5; about 15 mg/ml) in 36% (w/v) iodixanol and in water.

In Figure 3A the spectra of RI enhancers at high concentrations are presented. Very good spectral properties are shown by Ficoll^TM^ that can easily be used even around 300 nm. This is also possible with glycerol and sucrose but of less interest because of their limited ability to prevent light scattering (Figure 2). Very satisfactory and low absorbance is shown by Iodixanol above 400 nm. Below 400 nm a steep rise in absorbance is observed, although it is still manageable above 375 nm.

Two spectra of BSA are shown in Figure 3A, BSA 1 and BSA 2. Their spectra are quite different in the 375–450 region. Each curve corresponds to a BSA solution prepared from powder bought from the same manufacturer with the same catalogue number but different lot numbers. BSA 1 may be used down to 380 nm, although its spectral properties are less attractive than those of iodixanol. The smooth absorbance increment of BSA 1 is easy to compensate for, even if the target substance may also absorb in this region. This contrasts with BSA 2, which shows a distinct peak at around 410 nm. Such a peak may easily interfere with the quantification if the reference cuvette does not have a perfectly matched BSA concentration or if the target product is in low concentration.

The peak seen around 410 nm in BSA 2 is probably a heme/hemoglobin-related contamination in that lot. The contamination could be very minor on a weight basis but becomes prominent because of the very high BSA concentration used (300 mg/ml). In fact, the observed peak in BSA 2 can be explained by a hemoglobin content of only 0.02%. The BSA grades used in this study are described as >98% pure by the manufacturer. There are various grades of BSA available, such as those listed as globin-free. However, they can be up to ten times more expensive, which makes the idea of using RI enhancers less attractive due to the rather large amounts used. Incidentally, immunoglobulins have also been tested as RI enhancers with positive results; however, once again, they are much more expensive to use.

At lower wavelengths, all cells have an intrinsically high absorbance, as shown by the two spectra in Figure 3B. Both curves were obtained from *E. coli* in a concentration of 15 mg/ml (OD_600_ = 13.5). In one spectrum, the cells are suspended in water leading to high absorbance readings due to scattering. In the other spectrum with 37% (w/v) iodixanol, the scattering is largely absent and the absorbance at 550–650 nm is less than 1% compared to the spectrum without iodixanol. At lower wavelengths, the iodixanol spectrum starts to rise steeply due to the presence of many absorbing species inside the cell such as flavoproteins, heme containing proteins, NADH and other cofactors/metabolites.

This means that measuring of a specific, biotechnologically interesting metabolite at low concentration may be difficult below 400 nm, regardless of the spectral properties of the suspension medium. On the other hand, it might be of interest to determine the total extent of metabolites and the redox status, in which case spectra with RI enhancers suitable for low wavelengths should be of interest (e.g., Ficoll^TM^400).

To facilitate the use of RI enhancers, there are also properties like viscosity and density to consider (Supplementary Material: 3. Viscosity, density, and refractive index). Ficoll^TM^ and glycerol have very high viscosity, sucrose and BSA have moderately high viscosity, and iodixanol has a viscosity close to that of water. This property is by no means trivial from a practical point of view, since it is difficult to mix small volumes of phases with widely different viscosities, as pointed out in the Methods section. The low viscosity of iodixanol makes it very convenient and easy to use. However, its high density combined with its low viscosity can cause layering in the measuring cuvette if insufficient mixing is done.

### Comparison between the RI enhancer method and the method based on cell lysis for spectrophotometric Hb determination

A common way of determining Hb production in a bioreactor is to take a sample, collect the cells by centrifugation, and then disintegrate them with ultrasound. The supernatant is then recovered after centrifuging of cell debris to determine the spectrum and measure the concentration of Hb using the correspondent extinction coefficient (Antonini and Brunori, 1971; Simons et al, 2018). Use of the RI matching principle allows the omission of these time-consuming cellular disintegration and debris removal steps and allows for a more direct assessment of Hb content, even if the basic principle of quantification is the same.

To check the agreement of the two approaches, samples from a 16 h cultivation were taken and assayed in triplicate with the two methods. Details of the procedures, spectra and quantitative data are shown in Supplementary Material: 6. Comparison with a conventional Hb assay.

In Supplementary Figure S4 are shown the spectra obtained by the two methods and in Supplementary Table S8 are presented the calculated Hb concentrations, expressed as mg Hb per liter cultivation volume.

The RI enhancer method gave a more consistent result (Supplementary Table S8), most probably due to the fewer and more easily conducted steps in the RI method. Furthermore, both enhancers, iodixanol and BSA gave quite comparable results, 142 and 137 mg/L, respectively. On the other hand, the conventional, ultrasound-based method gave a much lower value, 51 mg/L (Supplementary Figure S4A). This discrepancy is explained by the fact that the ultrasonic method does not lyse 100% of the cells and some of the hemoglobin stays in the pellet inside non-lysed cells as shown by the photo of a centrifuged sample (Supplementary Figure S5). This is also demonstrated with the RI enhancer method in Supplementary Figure S4B, where it is shown that the cell debris from the ultrasound method contained a considerable amount of non-released Hb. The total amount of Hb, released and non-released, was 91 mg/L (Supplementary Table S8), a concentration considerably lower than the concentration determined with the RI enhancer method. Furthermore, in addition to be more laborious and time taking, the ultrasonic method may lead to sputtering of material and losses due to degradation of Hb (Weissler, 1960), all factors contributing to the underestimation of the true Hb content.

It is interesting to note that the RI enhancer method could be used to pinpoint the problem with the standard ultrasonic method, namely the underestimation of Hb concentration. This also points to an additional use of the RI enhancer method. It may be used not only to follow the progress of a fermentation, but it can also be used to assess the efficiency of cell disintegration procedures.

### A practical application of RI enhancers: Following the progress of hemoglobin production at different 5-ALA levels

**FIGURE 4:**
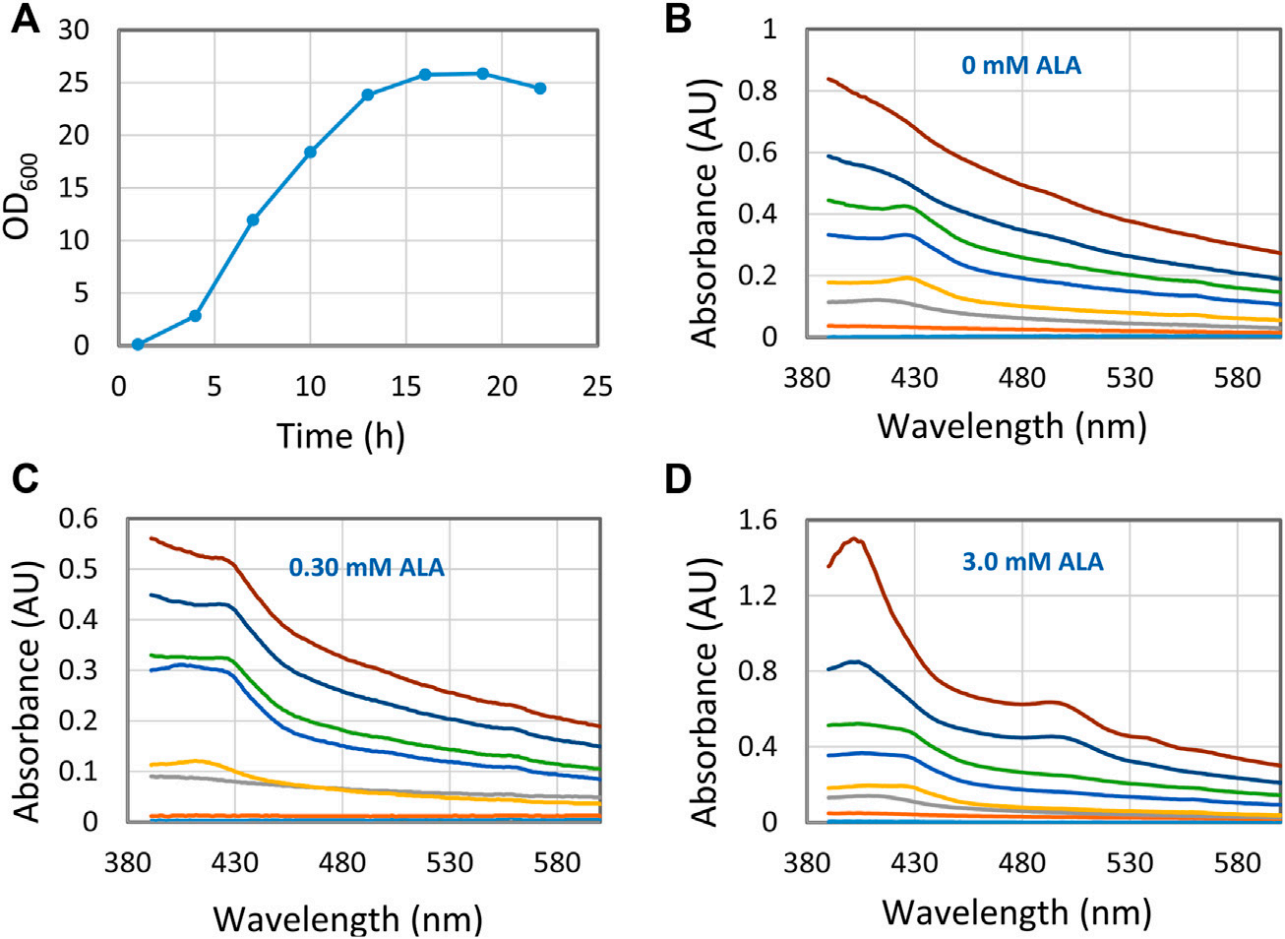
Hemoglobin/heme related compounds formed by *E. coli* at different 5-ALA (5-aminolevulinic acid) concentrations. **(A)** Growth curve with 0 mM 5-ALA. Essentially the same growth curve was observed for all experiments with different 5-ALA concentrations. **(B–D)** Spectra showing hemoglobin/heme related compounds formed at three 5-ALA concentrations. Samples were collected at 3 h intervals from 1 to 22 h. The 1-hour trace is close to the X-axis and the 22 h trace is at the top. For each occasion, 0.3 ml fermentation broth was sampled, the cells spun down, washed with Tris-HCl buffer, and suspended in 0.6 ml 30% (w/v) BSA.

When producing recombinant hemoglobin with the aim of using it as an artificial oxygen carrier the expensive precursor 5-aminolevulinic acid (5-ALA) is added. By using the RI enhancer method, we followed a set of fermentations with different concentrations of 5-ALA in near real-time, using conditions that are different from what the conventional protocol prescribes (Figure 4). Thus, a fermentation process was tested where 5-ALA and IPTG were added from the start of the cultivation. These conditions led to several different Hb species, species that interconverted during the fermentation, as evidenced by the shift of peak positions in the 400–430 nm region. Also, it was quickly concluded that 3.0 mM 5-ALA was the best concentration to obtain high concentrations of hemoglobin. Finally, the results at 0 mM 5-ALA did also tell us that *E. coli* is able to produce intrinsic heme and hemoglobin without the addition of external 5-ALA. Without the ultrasonic method these results would not have been that apparent, which shows the analytical strength of the RI enhancer method.

In a comparable manner, the RI enhancer method will be particularly useful to test other parameters such as additives and their concentration, temperature, pH, etc. to find out which are the best conditions for high protein expression.

The experiment illustrated in Figure 4, involved the intermittent automatic sampling and subsequent at line analysis. It should be mentioned that the use of refractive index enhancers may also facilitate a true in line analysis of intracellular compounds. A continuous or intermittent stream from the fermenter would be mixed with a stream of e.g., iodixanol and then passed through a flow-through cell in a UV/vis detector, providing real-time data of the Hb content.

## Conclusion

We have shown that RI enhancers may be used for convenient, rapid, and direct spectrophotometric analysis of intracellular target proteins. Especially iodixanol and BSA worked very well (low/medium viscosity, applicable for wavelengths down to 375–400 nm) making, for example, *E. coli* cells lose up to around 99% of their light scattering properties, thus greatly facilitating spectral investigations. The method allows fermentations to be followed in near real-time and should be especially welcome when developing new fermentation protocols where many analyses are needed to ensure optimal conditions. The method is also useful when checking the efficiency of cell disintegration in purification protocols.

BSA at 30% (w/v) or iodixanol at 36% (w/v) were found to be suitable for standard conditions. However, it was observed that for optimal conditions the concentration was dependent on the fermentation time–longer fermentation times required slightly higher concentration of refractive index enhancer to obtain minimum light scattering. BSA, being a high molecular compound/protein, will interfere minimally with the suspended cells. This may be an advantage compared to iodixanol, which on the other hand is easier to handle (low viscosity, better spectral properties).

All data in this report refer explicitly to the bacterium *E. coli.* Similar results were also obtained with the yeasts *Saccharomyces cerevisiae* and *Pichia pastoris* (Data not shown).

## Data Availability

The raw data supporting the conclusions of this article will be made available by the authors, without undue reservation.
