# Supplementary material for: Seeing through cells: Rapid measurement of intracellular target proteins
Source: Front Bioeng Biotechnol. 2022 Oct 3;10:996224. doi: 10.3389/fbioe.2022.996224 (PMC9574089; doi:10.3389/fbioe.2022.996224)
Supplement: Supplementary file 1 [file DataSheet1.pdf]

SUPPLEMENTARY MATERIAL

The supplementary material includes the following entries:

page
1. Special chemicals. 1
2. Cell cultivation. 1
3. Viscosity, density and refractive index. 1
4. Preparation and use of RI enhancer solutions 2
5. Cultivation time – shift of minimum. 7
6. Comparison with a conventional Hb assay 9

1. Special chemicals

Isopropyl thiogalactoside (IPTG), carbenicillin, 5-aminolevulinic acid (5-ALA) and several items of bovine serum albumin, BSA, (A7906 Lot #SLCH3825, A7030 Lot #SLCF3531, A7638 Lot #SLBW3326) were obtained from Sigma-Aldrich (Merck Darmstadt, Germany), Ficoll™400 from Pharmacia (Cytiva, Uppsala, Sweden) and iodixanol, 60 % w/w (Optiprep™) from Stemcells Technologies, Vancouver, Canada.

2. Cell cultivation

Escherichia coli BL21-DE3 cells expressing hemoglobins were cultivated as described by Leiva Eriksson et al. 2019. In short, cells containing the gene of a phytoglobin in an expression vector were grown in sterile TB medium containing 100 mg/mL carbenicillin. The gene expression was induced after 3h of growth by adding 0.5 mM IPTG and 0.4 mM 5-ALA. Harvesting of the cells was normally done around 16 h after induction..

In some experiments the progress of the fermentation was followed. To that end samples (3-5 ml) were withdrawn at selected intervals via a Teflon tubing attached to a peristaltic pump activated by a timer. The withdrawn samples were collected and stored in a cooled fraction collector until analyzed. The samples were usually analyzed with respect to OD_{600} (cell concentration), supernatant spectrum (after centrifugation), and cell spectrum (cell pellet suspended in refractive index enhancer).

3. Viscosity, density and refractive index

The viscosity was measured at room temperature in an Ostwald type osmometer (capillary flow). The viscosity data in the table are given relative to that of water. The densities were measured at room temperature with a 10 ml pycnometer and checked against literature data when available. The refractive index data were obtained at room temperature with a digital instrument (Pocket refractometer 3850, Atago Co, Japan). The data were checked against literature data when available. **Supplementary Table 1** gives data for some significant concentrations.

	Concentration (mg/mL)	Density (g/mL)	Relative viscosity	Refractive index
**Water**		1.00	1	1.333
**BSA**	300	1.08	16	1.388
**Ficoll™ 400**	300	1.09	52	1.374
**Glycerol**	900	1.20	30	1.433
**Iodixanol**	360	1.12	4	1.391
**Sucrose**	650	1.24	16	1.426

Supplementary Table 1. Density, viscosity and refractive index for RI enhancer solutions

4. Preparation and use of RI enhancer solutions

A. Preparation of iodixanol solutions

A 60 % (w/v) stock solution was obtained from Stemcells Technologies, Vancouver, Canada.

Density 1.320.

Supplementary Table 2 gives recipes for the preparation of 1 ml samples of a range of iodixanol concentrations and their corresponding RI values.

Supplementary Table 2. Preparation of 1 ml iodixanol solutions

Desired iodixanol concentration		60% (w/v) stock solution	Buffer or water	RI
(% w/v)	(% w/w)	(µL)	(µL)	
60	50.0	1000.0	0.0	1.429
55	46.5	916.7	83.3	1.421
50	42.9	833.3	166.7	1.413
45	39.1	750.0	250.0	1.405
42	36.8	700.0	300.0	1.400
40	35.3	666.7	333.3	1.397
38	33.7	633.3	366.7	1.394
36	32.1	600.0	400.0	1.391
34	30.5	566.7	433.3	1.388
30	27.3	500.0	500.0	1.381
20	18.8	333.3	666.7	1.365
10	9.7	166.7	833.3	1.349
0	0.0	0.0	1000.0	1.333

B. Preparation of BSA solutions

A 35 % (w/w) stock solution was prepared by weighing 17.50 g BSA in a 100 ml wide neck flask and then adding 32.50 g of water and placing the flask on a rocking table for several hours at room temperature. The obtained homogeneous solution contained an abundance of air bubbles, which largely disappeared after a couple of additional hours on the bench. This solution had a density at room temperature of 1.101 g/mL as measured by a 10 ml pycnometer, a value in agreement with calculations using the partial specific volume for BSA (0.739 ml/g).

Supplementary Table 3 gives recipes for the preparation of 1 ml samples of a range of BSA concentrations and their corresponding RI values.

Supplementary Table 3. Preparation of 1 ml BSA solutions

Desired BSA concentration		35 % (w/w) stock solution	Buffer or water	RI
(% w/v)	(% w/w)	(µL)	(µL)	
38	34.7	989.7	10.3	1.405
36	33.0	937.6	62.4	1.401
34	31.3	885.5	114.5	1.397
32	29.6	833.4	166.6	1.393
30	27.9	781.4	218.6	1.390
28	26.2	729.3	270.7	1.386
26	24.4	677.2	322.8	1.382
24	22.6	625.1	374.9	1.378
20	19.0	520.9	479.1	1.371
10	9.8	260.5	739.5	1.352
0	0.0	0.0	1000.0	1.333

C. Spectrophotometric measurements of cell suspensions

The spectrophotometric measurements were carried out according to a **standard procedure** where the cells were mixed with a RI enhancer solution having suitable concentration followed by spectrophotometric measurements. To test an additional RI enhancer concentration the whole procedure was repeated. The standard procedure was used in two variants: **variant I,** utilizing a centrifuged cell pellet and **variant II,** utilizing a cell suspension. Variant I is also presented in the main text, variant II is not.

An **alternative procedure (sequential addition*)*** was convenient to use when not knowing the optimal concentration of the RI enhancer. Here the cells were mixed with a strong RI enhancer solution and OD_{600} measured. Subsequently, a small, precalculated amount of water/buffer was added to give a new, known concentration of the RI enhancer. The cuvette content was mixed and OD_{600} again recorded and so on. Below follows a description of the **standard procedure** in its two variants and the **alternative procedure**.

Standard procedure, variant I

The bacterial cell suspension (0.50-2.00 ml) was centrifuged (2.5 min; 5,000 x g). The supernatant was removed, and the pellet suspended in 1.5 ml wash buffer (0.05 M Tris-HCl, pH 8.0). The cells were again spun down and the wash solution carefully removed. The pellet was subsequently suspended in 0.50 ml RI enhancer, e.g., 30 % (w/v) BSA or 36 % (w/v) iodixanol using a vibromixer (Lab-Line Instruments, IL, USA). **Supplementary Tables 2 and 3** give mixing data and RI for the resulting solutions. The volume of the pelleted bacteria and the volume of interstitial fluid was disregarded as being very small compared to the volume of the other ingredients. It was important to control that the pellet had been completely resuspended. The wash step in this procedure may be omitted.

The cell suspension was then immediately transferred to a cuvette (1.5 ml PMMA plastic cuvettes) for spectrophotometric measurements. To allow any gas bubbles to disappear from viscous solutions a few minutes waiting time was observed.

Standard procedure, variant II

Variant II is very similar to variant I except that a bacteria suspension is used instead of pelleted bacteria. The use of a suspension makes it also possible to carry out the mixing in the measuring cuvette. However, mixing in the cuvette is not a trivial operation since it involves the difficult mixing of a viscous component (BSA) or a high-density component (iodixanol) with low density/low viscosity water. A preferred way was to use a flat spatula and also to use a bright light source in order to observe any remaining Schlieren patterns indicating insufficient mixing, which would lead to misleading results. The use of a suspension obviously contributed to the volume of the mixture, thus changing the concentration of the RI enhancer. In **Supplementary Tables 4 and 5** two mixing examples are given, one for BSA and one for iodixanol. The total volume in the table is 0.5 ml which is a volume suitable for 1.5 ml plastic cuvettes, provided the cuvette is adjusted in height to match the spectrophotometer beam. Even smaller volumes are feasible with a proper cuvette.

Supplementary Table 4. Mixing cells and iodixanol in the cuvette – 0.5 ml volume

Desired iodixanol concentration		60% (w/v) iodixanol stock	Buffer or water	Bacteria suspen-sion	Relative bacterial dilution	RI
(% w/v)	(% w/w)	(µL)	(µL)	(µL)	(times)	
44	38.4	366.7	33.3	100	1.00	1.403
42	36.8	350.0	50.0	100	1.00	1.400
40	35.3	333.3	66.7	100	1.00	1.397
38	33.7	316.7	83.3	100	1.00	1.394
36	32.1	300.0	100.0	100	1.00	1.391
34	30.5	283.3	116.7	100	1.00	1.388
32	28.9	266.7	133.3	100	1.00	1.384
30	27.3	250.0	200.0	50	1.00	1.381
20	18.8	166.7	283.3	50	2.00	1.365
10	9.7	83.3	406.7	10	10.00	1.349
0	0.0	0.0	495.0	5	20.00	1.333

Supplementary Table 5. Mixing cells and BSA in the cuvette – 0.5 ml volume

Desired BSA concentration		35 % (w/w) BSA stock solution	Buffer or water	Bacterial suspen-sion	Relative bacterial dilution	RI
(% w/v)	(% w/w)	(µL)	(µL)	(µL)	(times)	
33	30.5	429.7	0.3	70	1.00	1.395
32	29.6	416.7	13.3	70	1.00	1.393
30	27.9	390.7	39.3	70	1.00	1.390
28	26.2	364.6	65.4	70	1.00	1.386
26	24.4	338.6	91.4	70	1.00	1.382
24	22.6	312.5	117.5	70	1.00	1.378
20	19.0	260.5	219.5	20	3.50	1.371
10	9.8	130.2	359.8	10	7.00	1.352
0	0.0	0.0	495.0	5	14.00	1.333

Alternative procedure (sequential addition)

To determine the approximate optimum concentration of a RI enhancer, it was convenient to carry out all mixing operations in the measuring cuvette, using a sequential dilution approach. To this end, bacterial cells were mixed with a concentrated RI enhancer and OD_{600} measured. Subsequently, a small, precalculated amount of water/buffer was added to give a new, known concentration. The cuvette content was mixed and OD_{600} again recorded and so on. This sequential dilution initially diminished the OD_{600}, the wanted minimum was reached, and then the OD_{600} again started to increase. This alternative procedure, although convenient to use, has the drawback that the cells are initially contacted with a too strong RI enhancer concentration, which may influence the cells, giving temporarily slightly skewed OD_{600} values.
The mixing in the cuvette is not a trivial operation since it involves the difficult mixing of a viscous component (BSA) or a high-density component (iodixanol) with low density/low viscosity water. A preferred way was to use a flat spatula and also to use a bright light source in order to observe any remaining Schlieren patterns indicating insufficient mixing which would lead to misleading results. After several water/buffer additions the viscosity and density differences became small and normal cuvette mixing was made (parafilm + inverting).

In **Supplementary tables 6 and 7** examples of two mixing protocols are given, one for iodixanol and one for BSA.

Supplementary Table 6. Mixing cells and iodixanol. Sequential addition of water/buffer

Desired Iodixanol Concentration		60 % (w/v) iodix. stock	Bacteria suspen-sion	Incre-mental buffer or water	Total volume	Relative bacterial dilution	RI
(% w/v)	(% w/w)	(µL)	(µL)	(µL)	(µL)	(times)	
48	41.4	400	100	0	500.0	1	1.410
46	39.9			21.7	521.7	1.04	1.407
44	38.4			23.7	545.5	1.09	1.403
42	36.8			26.0	571.4	1.14	1.400
40	35.3			28.6	600.0	1.20	1.397
38	33.7			31.6	631.6	1.26	1.394
36	32.1			35.1	666.7	1.33	1.391
34	30.5			39.2	705.9	1.41	1.388
32	28.9			44.1	750.0	1.50	1.384
30	27.3			50.0	800.0	1.60	1.381
20	18.8			400.0	1200.0	2.40	1.365

Supplementary Table 7. Mixing cells and BSA. Sequential addition of water/buffer

Desired BSA Concentration		60 % (w/v) BSA stock	Bacteria suspen-sion	Incre-mental buffer or water	Total volume	Relative bacterial dilution	RI
(% w/v)	(% w/w)	(µL)	(µL)	(µL)	(µL)	(times)	
33	30.5	430	70	0.3	500.3	1	1.395
32	29.6			15.6	515.6	1.03	1.393
30	27.9			34.4	550.0	1.10	1.390
28	26.2			39.3	589.3	1.18	1.386
26	24.4			45.3	634.6	1.27	1.382
24	22.6			52.9	687.5	1.38	1.378
20	19.0			137.5	825.0	1.65	1.371
10	9.8			825.0	1650.0	3.30	1.352

5. Cultivation time – shift of minimum

Cultivation time slightly influenced the concentration of the enhancer at which minimum light scattering was obtained. The experiment described here illustrates this. *E. coli* was cultivated under standard conditions for 19 hours. At even time intervals samples were collected and analyzed. OD_{600} was measured to get a growth curve as shown in **Supplementary Figure 1**.

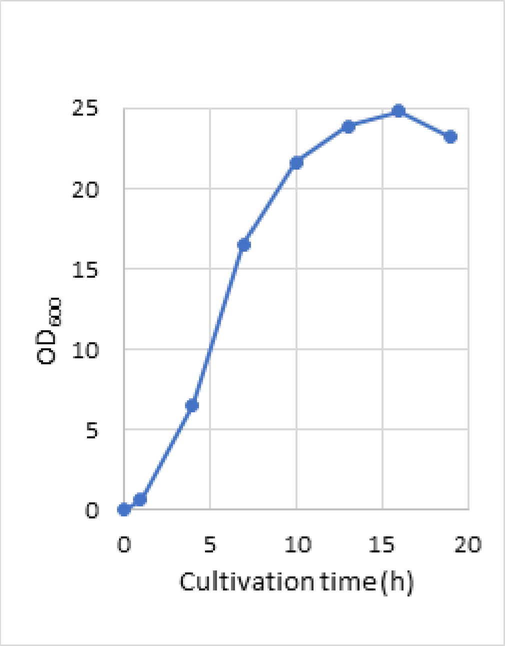

Supplementary Figure 1. Growth curve constructed from OD_{600} versus time

Light scattering properties were subsequently checked by suspending centrifuged cells in different concentrations of BSA or iodixanol and subsequently measuring the OD_{600}. The results for samples taken at 4 h, 10 h and 19 h are presented in **Supplementary Figure 2**.

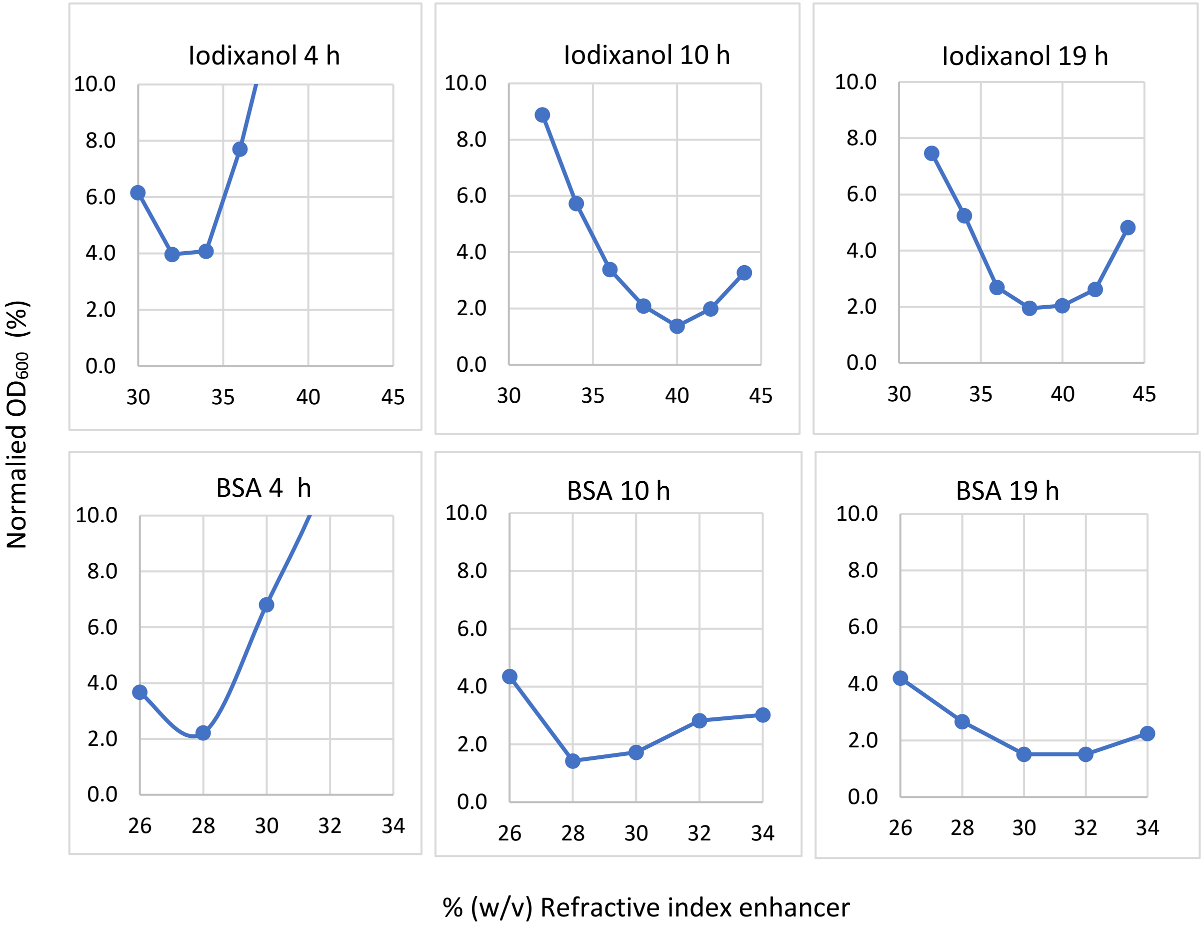

Supplementary Figure 2. Scattering curves with BSA and with iodixanol after 4, 10 and 19 hours of cultivation. *The different concentrations of RI enhancers used were obtained via the sequential dilution procedure (Methods section). Only concentrations rather close to the expected minimum were tested. The obtained OD_{600} readings were in several cases very low (down to 0.020 AU) meaning a lower accuracy. The readings have been normalized (a sample without refractive index enhancer has OD_{600} = 100 %).*

The minima from all sampling times are presented in **Supplementary Figure 3.** For BSA the minimum increased from about 28 % (w/v) to about 31 % /w/v), whereas for iodixanol the minimum increased from about 33 % (w/v) to about 40 % (w/v). The increase probably reflects inherently denser cells and perhaps also a higher permeability, suggested by the more pronounced increase with the low molecular weight iodixanol.

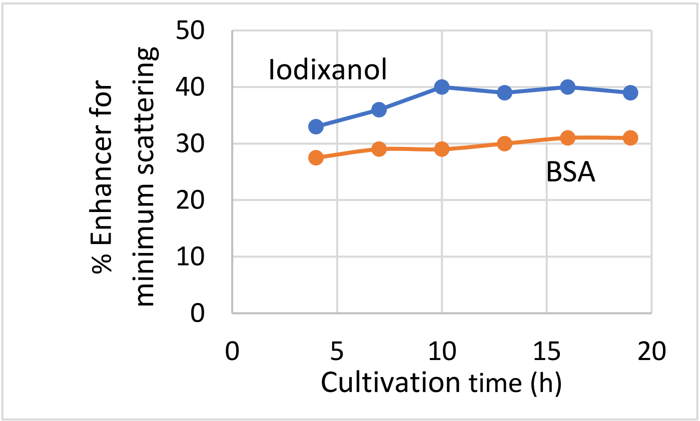

Supplementary Figure 3. *Enhancer concentration for minimum scattering as a function of cultivation time*

6. Comparison with a conventional Hb assay

The conventional way of determining Hb content (via ultrasonic cell disintegration) and the new RI enhancer method were compared. *E. coli* was cultivated and harvested 16 h after induction as described above in **Supplementary Materials 2**. The obtained cell suspension with OD_{600} = 14.7 was placed on ice and analyzed with the two methods.

Ultrasonic method
Three cell suspension samples (14 ml each) were centrifuged, and the weight of the wet cell pellet determined (250 mg). Twice the amount of Tris buffer was added (0.50 ml 0.05 mM pH 7.8) to each pellet to resuspend the cells. Then the cells were lysed with ultrasound (three cycles of 1 minute each at 30% amplitude and 20 % duty cycles, Branson Sonifier 250) in an ice bath. 1–2-minute waiting time was allowed between each ultrasound cycle to cool down the samples. Then, the homogenate was centrifuged, and the supernatant analyzed spectrophotometrically at 350 – 650 nm.
This procedure with triplicates took no less than 1 hour to carry out.

RI enhancer method
Three 250 µL samples of the cell suspension were spun down in 2 ml test tubes. The supernatant was carefully removed and 500 µL of 40 % iodixanol or 500 µL of 32 % BSA was added. Short treatment with a vibromixer suspended the cells. The test tubes were carefully inspected in order to detect any remaining non-suspended cells. The now clear cell suspension was analyzed spectrophotometrically at 350 to 650 nm. The reference cuvette contained 40 % (w/v) iodixanol or 32 % (w/v) BSA, respectively.
The procedure with triplicates for each RI enhancer tested (iodixanol and BSA) took about 30 min to carry out.

RI enhancer method to detect Hb not released by the ultrasonic method
The cell debris pellet generated by the ultrasonic method (see **Suppl. Fig. 5**) was suspended in 2.0 mlTris buffer. A sample from this suspension (50 µL) was mixed with 500 µL of Tris buffer (to dilute interstitial Hb), centrifuged and the supernatant carefully removed and discarded. Iodixanol (500 µL 40 % w/v) was added to the pellet. The cells were resuspended with a short treatment with a vibromixer. The test tube was carefully inspected in order to detect any remaining non-suspended cells. The now clear cell suspension was analyzed spectrophotometrically at 350 to 650 nm. The reference cuvette contained 40 % (w/v) iodixanol.

Evaluation of spectra
Hb content was determined by measuring the peak height. As baseline for peak measuring the tangent to the spectral curve was used at 460 or 475 nm. The use of the lower wavelength compensates for a higher background caused by contaminating chromophores. The drawing of a tangent and measuring of the peak height may be done manually but was here carried out automatically by an Excel routine after picking the appropriate point for the tangent. The Hb concentration calculation was also carried out by Excel, taking into account the concentration/dilution steps as described by the procedures given above. The spectra showed peaks at around 415 nm consistent with CO-bound Hb (maximum at 416 nm), and with minor contributions from oxidized Hb (maximum at 410 nm) (**Leiva Eriksson et al., 2019**). The concentration of the ferrous CO (carbonmonoxyHb) bound form of Hb was calculated using an extinction coefficient at 416 nm of 101.4 $mM^{-1}.cm^{-1}$ **(NLE's unpublished data)**. The results from the spectral data were presented as mg Hb per liter cultivation volume.

Results of the comparison
In **Suppl. Fig. 4, panels A-D** are shown the spectra obtained by the two methods and the calculated Hb concentrations expressed as mg Hb per liter of cultivation volume (averages of the triplicates). The RI enhancer method gave triplicates with very little spread as seen in **Suppl. Table 8**, presumedly due to few and easily conducted steps in the method. Furthermore, both iodixanol and BSA gave quite similar results, 142 and 137 mg/L.

Remarkable is that the conventional, ultrasound-based method gave a much lower value, 51 mg/L (**A**). A partial explanation is given in panel **B and Suppl Fig. 5**. Here is shown that the cell debris obtained with the ultrasound method contained a considerable amount of bound Hb. The sum of Hb in **A** and **B** is 91 mg/L (**Suppl. Table 8**), still lower than shown by the RI enhancer method. At least part of this discrepancy may be explained by the fact that the ultrasonic method is more laborious, may lead to sputtering and losses, and possibly that the ultrasound treatment degrades Hb or cause heme losses **(Weissler 1960)**.

It is interesting to note that the RI enhancer method could be used to pinpoint the problem with the standard ultrasonic method, namely its poor Hb release. This also points to an additional use of the RI enhancer method. It may be used not only to follow the progress of a fermentation, but it could also be used to check the efficiency of cell disintegration procedures.

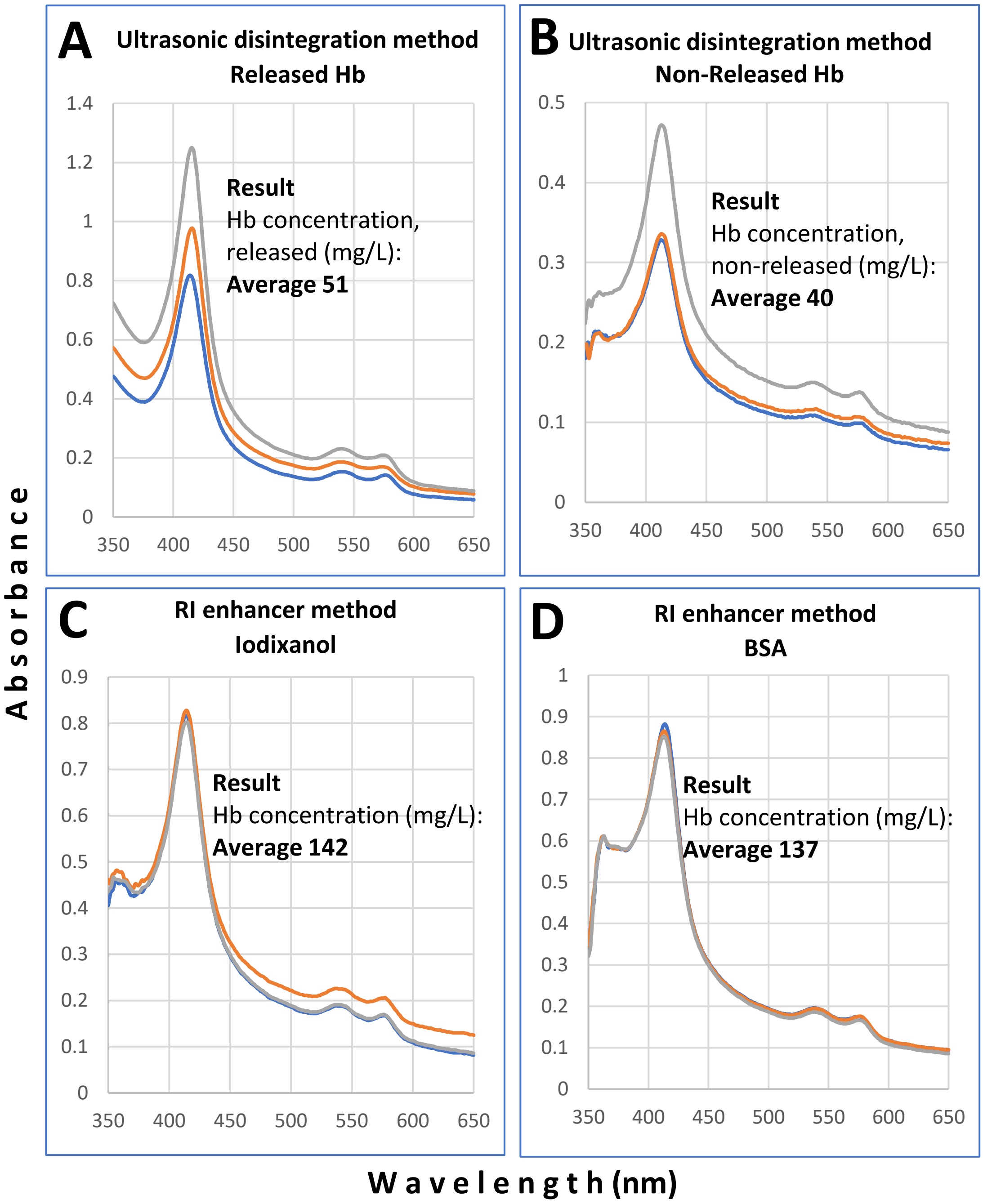

Supplementary Figure 4. *Comparison of the ultrasonic disintegration method and the RI enhancer method for the determination of Hb. Panel A shows the Hb released by the ultrasonic method. B shows the Hb remaining in the cell debris after the ultrasonic treatment (measured by the RI method), Panels C and D shows the results with the RI method using iodixanol (C) and BSA (D).*

Supplementary Table 8. Quantitative data obtained from Supplementary Figure 4.
The data is expressed in mg Hb per liter fermentation volume

Panel in Figure 4	Method	Triplicates			Average	Standard deviation
**A**	Ultrasonic, released Hb	40.9	49.3	63.6	51.3	±11.5
**B**	Ultrasonic, non-released Hb (measured via RI method)	33.8	34.3	51.8	40.0	±10.2
**C**	RI enhancer, iodixanol	145.8	141.2	138.5	141.8	±3.7
**D**	RI enhancer, BSA	132.5	139.3	139.5	137.1	±4.0

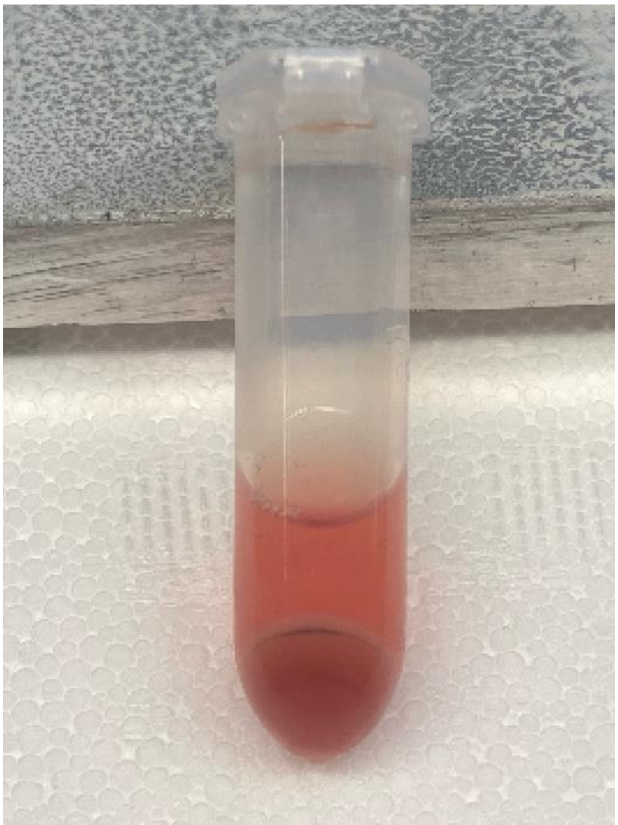

Supplementary Figure 5. Ultrasonic treatment. Released and non-released Hb.

The photo shows a centrifuged sample of phytoglobin producing E. coli after ultrasonic treatment. Note that not only the supernatant but also the pellet is strongly colored by Hb, proving that only a part of the Hb has been released into the supernatant. The non-released Hb is bound to intact cells and cell debris.
